# Adjuvant Use of PlasmaJet Device During Cytoreductive Surgery for Advanced-Stage Ovarian Cancer: Results of the PlaComOv-study, a Randomized Controlled Trial in The Netherlands

**DOI:** 10.1245/s10434-022-11763-2

**Published:** 2022-05-13

**Authors:** G. M. Nieuwenhuyzen-de Boer, W. Hofhuis, N. Reesink-Peters, S. Willemsen, I. A. Boere, I. G. Schoots, J. M. J. Piek, L. N. Hofman, J. J. Beltman, W. J. van Driel, H. M. J. Werner, A. Baalbergen, A. M. L. D. van Haaften-de Jong, M. Dorman, L. Haans, I. Nedelcu, P. C. Ewing-Graham, H. J. van Beekhuizen

**Affiliations:** 1 Department of Gynecologic Oncology, Erasmus MC Cancer Institute, University Medical Center Rotterdam, Rotterdam, The Netherlands; 2 Department of Obstetrics and Gynecology, Franciscus Gasthuis and Vlietland, Rotterdam, The Netherlands; 3 Department of Obstetrics and Gynecology, Medisch Spectrum Twente, Enschede, The Netherlands; 4 Department of Epidemiology, Erasmus MC, Rotterdam, The Netherlands; 5 Department of Biostatistics, Erasmus MC, Rotterdam, The Netherlands; 6 Department of Medical Oncology, Erasmus MC Cancer Institute, University Medical Center Rotterdam, Rotterdam, The Netherlands; 7 Department of Radiology and Nuclear Medicine, Erasmus MC Cancer Institute, University Medical Center Rotterdam, Rotterdam, The Netherlands; 8 Department of Obstetrics and Gynecology, Catharina Cancer Institute, Eindhoven, The Netherlands; 9 Department of Obstetrics and Gynecology, Albert Schweitzer Hospital, Dordrecht, The Netherlands; 10 Department of Obstetrics and Gynecology, Leiden University Medical Centre, Leiden, The Netherlands; 11 Department of Gynecology, Center of Gynecological Oncology Amsterdam, Netherlands Cancer Institute, Amsterdam, The Netherlands; 12 Department of Obstetrics and Gynecology, GROW, School for Oncology and Developmental Biology, Maastricht University Medical Centre, Maastricht, The Netherlands; 13 Department of Obstetrics and Gynecology, Reinier de Graaf Gasthuis, Delft, The Netherlands; 14 Department of Obstetrics and Gynecology, Haga Hospital, The Hague, The Netherlands; 15 Department of Obstetrics and Gynecology, Bravis Hospital, Bergen op Zoom, The Netherlands; 16 Department of Obstetrics and Gynecology, Haags Medical Centre, The Hague, The Netherlands; 17 Department of Obstetrics and Gynecology, Groene Hart Hospital, Gouda, The Netherlands; 18 Department of Pathology, Erasmus MC Cancer Institute, Rotterdam, The Netherlands

## Abstract

**Objective:**

Standard surgical treatment of advanced-stage ovarian carcinoma with electrosurgery cannot always result in complete cytoreductive surgery (CRS), especially when many small metastases are found on the mesentery and intestinal surface. We investigated whether adjuvant use of a neutral argon plasma device can help increase the complete cytoreduction rate.

**Patients and Methods:**

327 patients with FIGO stage IIIB–IV epithelial ovarian cancer (EOC) who underwent primary or interval CRS were randomized to either surgery with neutral argon plasma (PlasmaJet) (intervention) or without PlasmaJet (control group). The primary outcome was the percentage of complete CRS. The secondary outcomes were duration of surgery, blood loss, number of bowel resections and colostomies, hospitalization, 30-day morbidity, and quality of life (QoL).

**Results:**

Complete CRS was achieved in 119 patients (75.8%) in the intervention group and 115 patients (67.6%) in the control group (risk difference (RD) 8.2%, 95% confidence interval (CI) –0.021 to 0.181; *P* = 0.131). In a per-protocol analysis excluding patients with unresectable disease, complete CRS was obtained in 85.6% in the intervention group and 71.5% in the control group (RD 14.1%, 95% CI 0.042 to 0.235; *P* = 0.005). Patient-reported QoL at 6 months after surgery differed between groups in favor of PlasmaJet surgery (95% CI 0.455–8.350; *P* = 0.029). Other secondary outcomes did not differ significantly.

**Conclusions:**

Adjuvant use of PlasmaJet during CRS for advanced-stage ovarian cancer resulted in a significantly higher proportion of complete CRS in patients with resectable disease and higher QoL at 6 months after surgery. (Funded by ZonMw, Trial Register NL62035.078.17.)

**Trial Registration:**

Approved by the Medical Ethics Review Board of the Erasmus University Medical Center Rotterdam, the Netherlands, NL62035.078.17 on 20-11-2017. Recruitment started on 30-1-2018.

**Supplementary Information:**

The online version contains supplementary material available at 10.1245/s10434-022-11763-2.

Ovarian cancer is the eighth most common cancer in women, with nearly 314,000 new cases in 2020 worldwide.^1^ The most important independent prognostic factor for survival among patients with advanced-stage epithelial ovarian cancer (EOC) is completeness of cytoreductive surgery (CRS).^2–9^ Achieving complete CRS is difficult when many small tumor spots are found on the intestines and mesentery. The use of neutral argon plasma (PlasmaJet, Plasma Surgical, Inc, Roswell, GA), in addition to standard surgical instruments may help achieve complete CRS.^10–16^ We performed a study designed to assess whether adjuvant use of PlasmaJet would increase the proportion of complete CRS among patients with advanced-stage EOC.^17–20^

## Patients and Methods

### Trial Design

The PlaComOv study is a multicenter, single-blinded, randomized controlled superiority trial. The acronym “PlaComOv” already reveals the study aim: “Will the use of the PLAsmaJet® device improve the rate of COMplete cytoreductive surgery for advanced-stage OVarian cancer.”^17^

This trial compared the rates of complete CRS of patients with advanced EOC operated with standard use of electrocoagulation (control group) versus patients operated with adjuvant use of PlasmaJet (intervention group).

Patients from four gynecological oncology centers and nine centers specialized in ovarian cancer surgery in the Netherlands were randomized to either treatment arm. All hospitals had experience in CRS. A gyneco-oncologist from one of the oncology centers was always one of the surgeons. All surgeons were trained to perform operations with the PlasmaJet by following a course where theoretical knowledge of the PlasmaJet was discussed in detail, followed by operations on laboratory animals, concluding with an exam. During the cytoreductive surgery, someone with experience with PlasmaJet was always present.

For practical reasons, randomization was performed prior to surgery. Block randomization in a 1:1 ratio to either the intervention or control group was performed, with stratification according to suspected versus proven advanced-stage EOC, primary CRS (pCRS) versus interval CRS (iCRS), presence of peritoneal carcinomatosis based on preoperative computed tomography (CT) scan, and hyperthermic intraperitoneal chemotherapy (HIPEC) procedure.

All patients provided written informed consent and were blinded to the arm for which they were selected.

### Inclusion and Exclusion Criteria

Patients with suspected advanced-stage EOC, fallopian tube, or peritoneal carcinoma International Federation of Gynecology and Obstetrics (FIGO) stage IIIB–IV who were fit enough to undergo CRS and chemotherapy were eligible for inclusion. The surgical procedure was either pCRS or iCRS.^21,22^ Actual inclusion in the study was decided if advanced-stage EOC (FIGO IIIB–IV) was diagnosed during surgery. We excluded patients with recurrent disease, a nonepithelial, borderline ovarian tumor, or ovarian metastasis of another primary tumor, as well as patients who did not have surgery after randomization because of their condition.

HIPEC was introduced in the Netherlands in 2019.^23^ From 2019, all patients younger than 76 years of age with FIGO stage III EOC who underwent iCRS were eligible to receive an additional HIPEC procedure after complete or optimal CRS.

### Treatment

Preoperative workup consisted of physical examination and transvaginal ultrasonography. Serum measurement of cancer antigen 125 (CA-125) and carcinoembryonic antigen (CEA), a CT scan of the thorax/abdomen, and if possible a histological biopsy was taken. Workup findings were discussed preoperatively in a multidisciplinary tumor board.

Preoperative CT scans were reported systematically, and criteria were set for nonresectability of disease.^24–28^ Patients who met those criteria were scheduled for iCRS and received three courses of neoadjuvant chemotherapy (NACT). In case of response or stable disease on a CT scan after three cycles of chemotherapy, patients were eligible for iCRS.

Patients who had been included in this study but had incomplete primary CRS and thus received NACT to enable consecutive surgery remained in the treatment arm as allocated before primary surgery. For analyses, they were assigned to the iCRS group.

The standard chemotherapy regimen consisted of six cycles of intravenous carboplatin (area under the curve of 6 mg ml/min) and paclitaxel (175 mg/m^2^ body surface area) with a duration of 3 weeks for each cycle.^5^ In pCRS, all six cycles were given after surgery. In iCRS, in all cases, three cycles were given prior to and three cycles after surgery. In case of germline or tumor *BRCA* mutations, patients received maintenance of poly ADP ribose polymerase (PARP) inhibitor in accordance with standard of care as per April 2019.

Diagnostic laparoscopy was performed if the feasibility of complete CRS was doubted.

Surgery included total hysterectomy, bilateral salpingo-oophorectomy, omentectomy, and resection of all visible and palpable tumor. Complete, optimal, and suboptimal CRS was defined as described by the Gynecologic Oncology Group.^29,30^ Complete CRS was defined as surgery that resulted in no macroscopic disease (residual disease classification, R-1), optimal cytoreduction was defined as postoperative surgical residuum ≤ 1 cm in largest diameter (R-2), and suboptimal cytoreduction as residuum > 1 cm. Unresectable disease was defined as surgery intended to perform CRS but abandoned because tumor was irresectable.

Electrocoagulation, Harmonic Scalpel, Ligasure, scalpel, and scissors were used during conventional surgery to remove any visible tumor and to dissect tumor tissue on peritoneal surfaces.

In the intervention group, the PlasmaJet device could be used as an additional device. With the aim of objectifying surgical completeness, two gynecological oncologists blinded to the patient’s treatment arm allocation reviewed photographs from predesignated sites (pelvis, paracolic fossa, diaphragm, and small intestines) taken at the end of surgery.

At the end of each procedure in the intervention group, the gynecological oncologist filled in a questionnaire on the value of the contribution of the PlasmaJet to the surgical outcome.

All histology was coded, and the majority of the slides were reviewed by an experienced gyne-pathologist (P.E.E.-G.).

### End Points

The primary outcome was the rate of complete CRS. The secondary outcomes were duration of surgery, blood loss, length of hospitalization, bowel surgery, number of colostomies, complication rate (mortality and 30-day morbidity), and quality of life.

To study self-perceived health status, we asked patients to complete a questionnaire before surgery, and at 4 weeks and 6 months after surgery. The questionnaire consisted of two parts: a descriptive health classifier system on five dimensions: mobility, self-care, usual activities, pain/discomfort, and anxiety/depression (EQ-5D-5L), and a vertical visual analog scale (EQ-VAS).^31,32^

### Statistical Analysis

To demonstrate 15% more cases of complete CRS in the intervention group than in the control group (77% versus 62%) and setting the type I error (alpha) to 5% and type II error (beta) to 20%, we needed to enroll 294 patients. Assuming 12% loss to follow-up, 330 patients were required.

An intention-to-treat (ITT) analysis was performed with data of all included patients. A per-protocol analysis was performed with data of all patients who underwent CRS.

The primary outcome was calculated with a confidence interval based on the Wilson method. Group data were compared using a chi-squared test with continuity correction. The arms of the trial are compared using a generalized linear model with a binomial distribution and identity link adjusting for stratification factors. We further present an unadjusted risk difference together with a 95% confidence interval based on Newcombe’s method.

An exploratory subgroup analysis was performed in a subset of patients who underwent HIPEC and patients with ≥ 50 lesions in the abdomen (peritoneal carcinomatosis), which made complete CRS not easily feasible.

Continuous secondary outcomes (duration of surgery, duration of hospital stay, blood loss, and patient-reported quality of life on EQ-VAS and EQ-5D-5L questionnaires) were compared using *t*-tests; the discrete variables (complication rate, bowel surgery, and colostomies) were compared using chi-square tests with continuity correction, unless an expected count was less than five, in which case Fisher’s exact test was used.

*P*-value < 0.05 on a two-sided test was considered to indicate a significant difference. All analyses were performed using R 4.1 (Foundation for Statistical Computing Vienna, Austria). Multiplicity correction was not performed for this subgroup analysis.

## Results

### Patients

**Fig. 1:**
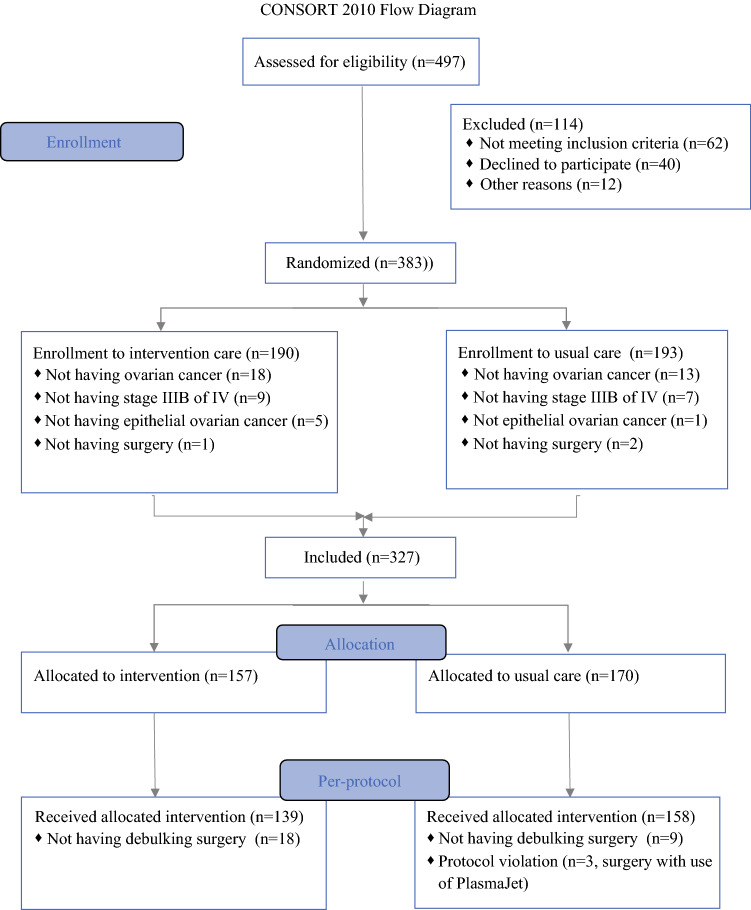
CONSORT 2010 flow diagram

From February 2018 through September 2020, a total of 383 patients were randomized: 190 to the intervention group and 193 to the control group. All had suspected or proven advanced-stage EOC (Fig. 1). Fifty-six patients had to be excluded. The clinical characteristics of the 327 included patients whose data were analyzed according to intention to treat are presented in Table 1. The characteristics are evenly distributed among the two groups.

**Table 1:** Patient characteristics

	Intention to treat		Per protocol	
	Intervention *N* = 157 (%)	Control *N* = 170 (%)	Intervention *N* = 139 (%)	Control *N* = 158 (%)
*Age (years)*				
Mean [SD]	66.1 [9.6]	65.1 [11.2]	65.8 (9.3)	64.9 (11.3)
Median [min, max]	67.6 [28.9, 81.3]	65.9 [20.3, 86.1]	66.9 [35.4, 81.2]	65.7 [20.3, 86.1]
*Parity (>AM 24 weeks)*				
0	22 (14.0)	34 (20.0)	20 (14.4)	32 (20.3)
1–2	99 (63.1)	83 (48.8)	88 (63.3)	76 (48.1)
> 3	34 (21.7)	49 (28.8)	29 (20.8)	46 (29.1)
*WHO performance status*				
0	82 (52.2)	90 (52.9)	76 (54.7)	85 (53.8)
1	56 (35.7)	53 (31.2)	46 (33.1)	50 (31.6)
2	9 (5.7)	8 (4.7)	7 (5.0)	5 (3.2)
3	2 (1.3)	5 (2.9)	2 (1.4)	5 (3.2)
4	1 (0.6)	0	1 (0.7)	0
*Body mass index (kg/m*^*2*^*)*				
Mean [SD]	24.8 [5.30]	25.7 [4.37]	24.7 [5.00]	25.9 [4.39]
Median [min, max]	24.0 [17.2, 57.1]	24.9 [17.3, 40.6]	24.2 [17.8, 57.1]	24.9 [17.3, 40.6]
Missing	0	1 (0.6)	0	1 (0.6)
*Ca-125 diagnosis (kU/l)*				
Mean [SD]	2250 [3710]	1810 [3500]	2220 [3640]	1790 [3530]
Median [min, max]	849 [5.0, 25,400]	776 [26.0, 31,600]	881 [5, 25,400]	776 [26, 31,600]
Missing	0	4 (2.4)	0	1 (0.6)
*Ca-125 preoperative (kU/l)*				
Mean [SD]	426 [1450]	319 [698]	452 [1540]	311 (690)
Median [min, max]	92.2 [6.0, 13,000]	72.0 [9.0, 5090]	94 [6, 13,000]	71 [26, 31600]
Missing	9 (5.7)	6 (3.5)	9 (6.5)	6 (3.8)
*CEA pre-operative (µg/l)*				
Mean [SD]	6.03 [31.2]	3.89 [10.5]	6.53 [33.3]	3.97 (10.8)
Median [min, max]	1.75 [0.1, 304]	1.6 [0, 93.0]	1.75 [0.1, 304]	1.6 [0, 93]
Missing	61 (38.9)	65 (38.2)	55 [39.6]	59 (37.3])
*Histology*				
Sereus adenocarcinoma	149 (94.9)	164 (96.5)	131 (94.2)	152 (96.2)
Mucinous adenocarcinoma	1 (0.6)	1 (0.6)	1 (0.7)	1 (0.6)
Endometroid adenocarcinoma	0	2 (1.2)	0	2 (1.3)
Clearcell adenocarcinoma	5 (3.2)	0	5 (3.6)	0
Mixed epithelial carcinoma	0	1 (0.6)	0	1 (0.6)
Carcinosarcoma	2 (1.3)	2 (1.2)	2 (1.4)	2 (1.3)
*FIGO stage*				
IIIB	11 (7.0)	11 (6.5)	11 (7.9)	10 (6.3)
IIIC30	96 (61.1)	109 (64.1)	85 (61.2)	103 (65.2)
IV	50 (31.8)	50 (29.4)	43 (30.9)	45 (28.5)
Primary CRS	20 (12.7)	25 (14.7)	20 (14.4)	22 (13.9)
Interval CRS	137 (87.3)	145 (85.3)	119 (85.6)	136 (86.1)
Suspicion peritoneal carcinomatosis on CT	111 (70.7)	113 (66.5)	96 (69.1)	106 (67.1)
HIPEC procedure	29 (18.5)	32 (18.8)	29 (20.9)	32 (20.3)

*CRS* Cytoreductive surgery, *SD* standard deviation

In 27 patients (8.3%), a laparotomy was performed but CRS was not performed because of unresectable disease. These 27 patients were not evenly distributed among the two groups, with 18 patients in the intervention group and 9 in the control group. Three others were left out of the analysis because of protocol violation: although they had been randomized to the control group, the PlasmaJet was still used during surgery (Table 1).

Forty-five patients (14.8%) underwent pCRS, and 282 patients (86.2%) iCRS. Twenty-six patients (8%) underwent a diagnostic laparoscopy prior to CRS, in which pCRS was possible in 12 patients. Fourteen patients started with NACT followed by iCRS, being evenly distributed among the groups.

### Surgical Outcomes

The intention-to-treat analysis showed that complete CRS was achieved in 75.8% (95% CI 0.685–0.813) of patients in the intervention group versus 67.6% (95% CI 0.603–0.742) in the control group (RD 8.2%, 95% CI –0.021 to 0.181; *P* = 0.131, adjusted for stratification factors, RD 9.1%, 95% CI –0.01 to 0.20; *P* = 0.070). Other surgery details are provided in Table 2.

**Table 2:** Surgical outcomes

	Intention to treat			Per protocol		
	Intervention *N* = 157 (%)	Control *N* = 170 (%)	*P*-value	Intervention *N* = 139 (%)	Control *N* = 158 (%)	*P*-value
*Surgical outcome*						
Complete	119 (75.8)	115 (67.6)	0.001	119 (85.6)	113 (71.5)	0.002
Optimal	12 (7.6)	38 (22.4)		12 (8.6)	38 (24.1)	
Suboptimal	8 (5.1)	8 (4.7)		8 (5.8)	7 (4.4)	
Unresectable	18 (11.5)	9 (5.3)		-	-	
Complete cytoreductive surgery YES	119 (75.8)	115 (67.6)	0.131	119 (85.6)	113 (71.5)	0.005
*Start of surgery*						
Primary CRS	20 (12.7)	25 (14.7)	0.722	20 (14.4)	22 (13.9)	1
Interval CRS	137 (87.3)	145 (85.3)		119 (85.6)	136 (86.1)	
*Operative time (min)*						
Mean [SD]	236 [126]	222 [110]	0.326	254 [121]	230 [109]	0.084
Median [min, max]	210 [29, 671]	194 [48, 595]		234 [45, 671]	202 [65, 595]	
Missing	6 (3.8)	4 (2.4)		5 (3.6)	3 (1.9)	
Abdominal drain	35 (22.3)	50 (29.4)	0.259	35 (25.2)	49 (31.0)	0.263
*Blood loss (ml)*						
Mean [SD]	923 [801]	956 [801]	0.712	1020 [803]	995 [805]	0.827
Median [min, max]	700 [0, 4300]	845 [0, 6000]		800 [50.0, 4300]	875 [0, 6000]	
Missing	4 (2.5)	1 (0.6)		4 (2.9)	0	
Transfusion during surgery	41 (26.1)	45 (26.5)	0.877	41 (29.5)	45 (28.5)	0.961
Colostomy	9 (5.7)	20 (11.8)	0.092	9 (6.5)	20 (12.7)	0.169
Intensive care postoperative	34 (21.7)	40 (23.5)	0.785	33 (23.7)	39 (24.7)	0.957
*Intensive care (days)*						
Mean [SD]	1.9 (1.9)	1.6 (0.9)	0.339	1.9 [1.9]	1.6 [0.9]	0.378
Median [min, max]	1.0 [1,11]	1.0 [1,5]		1.0 [1,11]	1.0 [1,5]	
*Hospitalization (days)*						
Mean [SD]	8.7 [6.5]	7.9 [6.4]	0.221	9.1 [6.7]	8.1 [6.6]	0.175
Median [min, max]	6.5 [2, 35]	6.0 [2, 51]		7.0 [2, 35]	6.0 [3, 51]	
Missing	3 (1.9)	0		3 (2.2)	0	
*Discharge*						
Home without nursing care	100 (63.7)	110 (64.7)	0.955	86 (61.9)	101 (63.9)	0.975
Home with nursing care	34 (21.7)	39 (22.9)		31 (22.3)	38 (24.1)	
Nursing home	4 (2.5)	3 (1.8)		3 (2.2)	3 (1.9)	
Rehabilitation center	2 (1.3)	2 (1.2)		2 (1.4)	2 (1.3)	
Hotel providing nursing care	9 (5.7)	12 (7.1)		9 (6.5)	10 (6.3)	
Hospice	1 (0.6)	0		1 (0.7)	0	
Death	0	0		0	0	

*CRS* cytoreductive surgery

In the per-protocol analysis, complete CRS was achieved in 85.6% (95% CI 0.788–0.905) of patients in the intervention group versus 71.5% (95% CI 0.640–0.780) in the control group (RD 14.1%; 95% CI 0.042–0.235; *P* = 0.005, adjusted for stratification factors, RD 14.0%, 95% CI 0.050–0.231; *P* = 0.003).

In case of pCRS (*n* = 42), complete CRS was achieved in 90.0% of patients in the intervention group versus 63.6% in the control group (RD 26.4%, 95% CI –0.032 to 0.506; *P* = 0.071, adjusted for stratification factors, RD 27.9%, 95% CI 0.057–0.522; *P* = 0.018). In case of iCRS (*n* = 255), complete CRS was achieved in 84.9% of patients in the intervention group versus 72.8% in the control group (RD 12.1%, 95% CI 0.014–0.222; *P* = 0.031, adjusted for stratification factors, RD 12.2%, 95% CI 0.024–0.218; *P* = 0.015) (Supplementary Table S1).

The median operating time in the intervention group was 33 min longer than in the control group (*P* = 0.056). As displayed in Supplementary Table S3, on subanalysis, operating time during CRS including HIPEC was longer than in the group without a HIPEC procedure. The median operating time during CRS including HIPEC was 392 min (intervention) versus 372 min (control group). The median operating time during CRS without HIPEC was 219 min (intervention) versus 193 min (control).

There was no significant difference in volume of blood loss and blood transfusion between the groups. The duration of postoperative hospital stay did not statistically significantly differ between the groups.

The number of colostomies was lower in the intervention group (6.5% versus 12.7%) but did not differ significantly (*P* = 0.169) (Table 2).

In the intervention group, nine women received a colostomy: six a permanent colostomy and three a temporary colostomy. In the control group, 20 women received a stoma: 8 a permanent colostomy, 11 a temporary colostomy, and 1 an ileostomy because the whole colon had to be removed. Twelve months after surgery, none of the women with a temporary colostomy had reversal of their colostomy.

Bowel surgery was performed in about 50% of the patients in both groups. The most common type of resection was rectosigmoid resection (*n* = 46, 15.7%). The type of surgical procedure (removal of the tumor from the bowel or resection of the organ) did not significantly differ between groups, except for rectal involvement. Rectal involvement was found in 52 of the 139 patients (37.5%) in the intervention group versus 45 of the 158 patients (28.5%) in the control group. To achieve complete CRS, the rectosigmoid was resected in 8 patients (5.8%) in the intervention group and 15 patients (9.5%) in the control group (*P* = 0.033) (Supplementary Table S2).

### Complications

The surgical complication rate did not significantly differ between the two groups (Table 3). A relaparotomy was performed in eight patients of the intervention group. No relaparotomy was related to the use of the PlasmaJet.

**Table 3:** Surgical complications within 30 days (per-protocol analysis)

	Intervention group *N* = 139 (%)	Control group *N* = 158 (%)	*p*-Value
Bowel laceration postoperative	2 (1.4)	1 (0.6)	0.597
*Bowel obstruction (ileus)*			
Conservative	11 (7.9)	14 (8.9)	1
Surgery	0	0	
*Surgical-site infection*			
Sepsis	0	4 (2.5)	0.127
Intraabdominal abscess	1 (0.7)	3 (1.9)	0.627
Urinary tract infection	8 (5.8)	7 (4.4)	0.735
Superficial wound infection	8 (5.7)	4 (2.5)	0.201
Relaparotomy*	8 (5.8)	3 (1.9)	0.143
*Medical complication*			
Cardiac	6 (4.3)	7 (4.4)	1
Venous thromboembolism	1 (0.7)	2 (1.3)	1
Deep venous embolism	1 (0.7)	2 (1.3)	1
Pulmonary embolism	2 (1.4)	3 (1.9)	1
Pulmonary failure	0	0	1
Pneumonia	2 (1.4)	10 (6.3)	0.072
Respiratory insufficiency	7 (5.0)	5 (3.1)	0.551
Renal failure	1 (0.7)	1 (0.6)	1
Ureter laceration	0	0	1
Gastric perforation	1 (0.7)	0	1
Anastomotic leakage	1 (0.7)	1 (0.6)	1
Stroke	1 (0.7)	0	1
Delirium	5 (3.6)	1 (0.6)	0.099
Death (within 30 days)	0	1 (0.6)	0.319

*Indications intervention group: anastomotic leakage (1), suspicion of anastomotic leakage (2), to continue and finish the interval debulking surgery (1), gastric perforation (1), pancreatic leakage (1), intraabdominal bleeding (1), pelvic abscess (1).

Control group: anastomotic leakage (1) and suspicion of anastomotic leakage (2)

A paralytic ileus developed in 8.5% of all cases, evenly distributed in both groups, and resolved with conservative treatment. Apart from a higher rate of postoperative pneumonia in the control group, there were no significant differences in postoperative complications within 30 days following surgery between the two groups.

The cumulative incidence of mortality within 30 days was 0.003%. One of the patients in the control group died at home the night after discharge from hospital. An autopsy was not performed.

#### HIPEC

A subset analysis was performed on data of 61 patients with FIGO stage III disease who underwent iCRS combined with HIPEC (Supplementary Table S3). This showed a higher percentage of complete CRS in the intervention group compared with the control group, which was not significant (96.6% compared to 81.2%, *P* = 0.106).

### Peritoneal Carcinomatosis

A subset analysis was performed in a group of patients with disseminated intraabdominal disease, called peritoneal carcinomatosis (Supplementary Table S4). This was defined as ≥ 50 metastatic lesions on either peritoneum, diaphragm, or mesentery. A total of 120 patients had ≥ 50 lesions. The rate of complete CRS of these patients was 72.2% in the intervention group versus 51.5% in the control group (RD 20.7%, 95% CI 0.020–0.373; *P* = 0.034).

### Use of PlasmaJet

In the intervention group, the PlasmaJet was used 104 times during surgery (75%). In 56 of all patients in the intervention group (41%), the gynecological oncologist gave their opinion on whether PlasmaJet was necessary or very useful to achieve complete CRS (Supplementary Table S5). In 12% of the procedures, PlasmaJet was regarded as necessary to achieve complete CRS.

Regarding the learning curve, expertise in using the PlasmaJet did not affect surgical outcome (surgical procedure 1–10 versus > 10) (Supplementary Table S6).

### Patient-Reported Outcomes

**Fig. 2:**
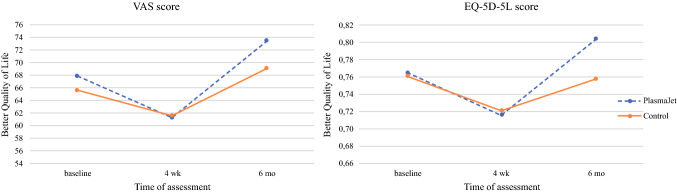
Quality of life results

Patients self-rated their health status before surgery (299 responders, 91.4%), and at 4 weeks (296 responders, 90.5%) and 6 months (262 responders, 80.1%) after surgery. Six months after surgery, patients in the intervention group (*n* = 120) reported a better health score (EQ-VAS 73.4) than the patients in the control group (*n* = 142) (EQ-VAS 69.0) (95% CI 0.455–8.350; *P* = 0.029). Six months after surgery, patients in the intervention group reported a mean EQ-5D-5L health state of 0.80 compared with 0.76 in the control group (95% CI 0.001–0.092; *P* = 0.049) (Fig. 2).

## Discussion

In this randomized, multicenter clinical trial on the effectiveness of the PlasmaJet device during CRS for advanced-staged EOC, surgery with adjuvant use of the PlasmaJet was associated with a significantly higher proportion of complete CRS in patients with resectable disease.

This benefit was even stronger in the subset analysis of patients with peritoneal carcinomatosis.

These results are consistent with previous results based on case series of patients with EOC and treated with PlasmaJet.^11–16^

A per-protocol analysis was performed in which 27 patients with unresectable disease were excluded, considering that the aim of the study was to examine the effectiveness of the use of the PlasmaJet during CRS.

In 12%, the gynecological oncologists indicated that the PlasmaJet was regarded as “necessary” to achieve complete CRS. In case of many small tumor spots at the small intestines, it is often not possible to remove all tumor lesions without bowel resection. In case of more than two to three anastomosis in often frail, elderly patients or in case of a large small-bowel resection that would lead to a short-bowel syndrome, all those small tumor spots cannot be removed without the help of the PlasmaJet. The same applies to many small tumor spots at the location of the small bowel mesentery. If this has to be removed using electrocoagulation, there is a greater chance of damage to the blood supply of the small intestine than with the use of the PlasmaJet.^34^ In this study, we see the benefit of the use of the PlasmaJet for surgical outcome even more strongly in the subset analysis of patients with peritoneal carcinomatosis (Supplementary Table S4).

In 29%, the gynecological oncologists indicated that it was “very useful” to use the PlasmaJet, mainly because the PlasmaJet simplifies the removal of lesions at the location of the diaphragm and peritoneum compared with electrocoagulation. Especially at the location of the diaphragm, the PlasmaJet has added value because it does not cause muscle contractions.

This study is a single-blind RCT. It was impossible to evaluate the completeness of surgery in a double-blind setting, as electrocoagulation and use of the PlasmaJet leave different scars. At the end of surgery, photographs were taken to objectively estimate the result of CRS. Two gynecological oncologists independently reviewed a number of the photographs. The final judgment was hampered by the fact that there was no overview of the complete abdomen and palpation was not feasible. Although this method resulted in a subjective interpretation, the conclusion on surgical outcome seems reliable. Given the high percentage of complete and optimal CRS, a postoperative CT scan would have had no added value, as it does not show small tumor volume.

None of the secondary outcomes differed significantly between the intervention group and the control group. Still, the duration of surgery with the adjuvant use of the PlasmaJet was 32 min longer (*P* = 0.084). More often, the use of the PlasmaJet made it possible to remove tumor lesions at vulnerable locations. Although this takes more time, a higher percentage of complete CRS can be reached.

Bowel surgery was performed in 50% of the patients in both groups. Patients in the intervention group had more frequent disease involvement of the surface of rectum and rectosigmoid. Disease at these sites was removed more often without the need for resection compared with the control group. Besides a lower proportion of bowel resections in the intervention group, the number of colostomies in the intervention group was lower than in the control group. A colostomy was created when there was no possibility to perform an anastomosis or when such an extensive resection was performed that the surgeon decided that the risk of anastomotic leakage was too high.

Table 2 and Supplementary Table S2 (per-protocol analysis) show that resection of the bowel was performed in 37 of 139 patients (26.6%) in the intervention group, of whom 9 got a colostomy (6.5%). Of all 37 patients with bowel surgery, 9 got a colostomy (24.3%).

In the control group, bowel resection was performed in 56 of 158 patients (35.4%), of whom 20 got a colostomy (12.7%). Of all 56 patients with bowel surgery, 20 got a colostomy (35.7%). This was not significantly different. Although this study was not powered for differences in bowel surgery, fewer colostomies in the intervention group is an important finding. Further research should demonstrate whether the use of PlasmaJet can avoid bowel surgery and colostomies.

Six months after surgery, patients in the intervention group reported a better health score than the patients in the control group. A possible explanation could be the lower percentage of colostomies in the intervention group (9 versus 20). Another explanation for the more favorable health scores in the intervention group is perhaps the long-term protective effect of PlasmaJet, which results in less tissue damage than coagulation.^10,33,34^

In conclusion, in this trial, adjuvant use of the PlasmaJet during CRS for advanced-stage EOC resulted in a higher proportion of complete CRS and is significantly associated with a better patient-reported outcome at 6 months after surgery.

Considering that the surgical outcome has important impact on both PFS and OS,^3,6,7^ we recommend considering the use of the PlasmaJet during CRS to remove all visible tumor when many small metastases at vulnerable locations are found. Still, survival data need to mature to assess the effect on PFS and OS outcomes.

## Supplementary Information

Below is the link to the electronic supplementary material.

Supplementary file1 (DOCX 14 kb)

Supplementary file2 (DOCX 14 kb)

Supplementary file3 (DOCX 14 kb)

Supplementary file4 (DOCX 13 kb)

Supplementary file5 (DOCX 13 kb)

Supplementary file6 (DOCX 13 kb)

## Data Availability

Research data at Erasmus MC is generated, stored, and made accessible in accordance with legal, academic, and ethical requirements. This study, and all persons involved, have knowledge of and comply with the most recent version of the Erasmus MC Research Code, which complies with all current laws and regulations. Data will be handled practicing the FAIR principles (Findable, Accessible, Interoperable, and Reusable) according to the Handbook for Adequate Natural Data Stewardship (HANDS) developed by the Federation of Dutch UMCs. All research data are handled confidentially in accordance with legislation and conditions imposed by The Dutch Data Protection Authority. The research data from this study are stored in a long-term archive on secured network servers, with regular back-up and limited access. In accordance with the Netherlands Code of Conduct for Scientific Practice, raw data are stored for a period of at least 10 years. Permission for third persons to access the data will only be granted by the PI on certain conditions.
