# Supplementary material for: Adjuvant Use of PlasmaJet Device During Cytoreductive Surgery for Advanced-Stage Ovarian Cancer: Results of the PlaComOv-study, a Randomized Controlled Trial in The Netherlands
Source: Ann Surg Oncol. 2022 May 13;29(8):4833–43. doi: 10.1245/s10434-022-11763-2 (PMC9246793; doi:10.1245/s10434-022-11763-2)
Supplement: Supplementary file 1 — Supplementary file1 (DOCX 14 kb) [file 10434_2022_11763_MOESM1_ESM.docx]

Table S1. Surgical outcome in case of primary cytoreductive surgery or interval cytoreductive surgery

|  | Primary CRS | |  | Interval CRS | |  |
| --- | --- | --- | --- | --- | --- | --- |
|  | **Intervention**  **n=20 (%)** | **Control n=22 (%)** | P.overall | **Intervention n=119 (%)** | **Control n=136 (%)** | P.overall |
| Complete cytoreduction |  |  | 0.071 |  |  | 0.029 |
| YES | 18 (90.0) | 14 (63.6) |  | 101 (84.9) | 99 (72.8) |  |
| NO | 2 (10.0) | 8 (36.4) |  | 18 (15.1) | 37 (27.2) |  |

CRS=Cytoreductive surgery
