# Supplementary material for: Adjuvant Use of PlasmaJet Device During Cytoreductive Surgery for Advanced-Stage Ovarian Cancer: Results of the PlaComOv-study, a Randomized Controlled Trial in The Netherlands
Source: Ann Surg Oncol. 2022 May 13;29(8):4833–43. doi: 10.1245/s10434-022-11763-2 (PMC9246793; doi:10.1245/s10434-022-11763-2)
Supplement: Supplementary file 2 — Supplementary file2 (DOCX 14 kb) [file 10434_2022_11763_MOESM2_ESM.docx]

Table S2. Bowel surgery during cytoreductive surgery

| Tumor site | Intervention  n=139 (%) | Control  n=158 (%) | P-value |
| --- | --- | --- | --- |
| Rectum |  |  |  |
| No tumor | 84 (60.4) | 108 (68.4) | 0.033 |
| Removal tumor | 44 (31.7) | 30 (19.0) |  |
| Resection organ | 8 (5.8) | 15 (9.5) |  |
|  |  |  |  |
| Recto-sigmoid |  |  |  |
| No tumor | 71 (51.1) | 85 (53.8) | 0.224 |
| Removal tumor | 49 (35.3) | 42 (26.6) |  |
| Resection organ | 18 (12.9) | 28 (17.7) |  |
|  |  |  |  |
| Cecum |  |  |  |
| No tumor | 102 (73.4) | 109 (69.0) | 0.779 |
| Removal tumor | 30 (21.6) | 39 (24.7) |  |
| Resection organ | 6 (4.3) | 7 (4.4) |  |
|  |  |  |  |
| Appendix |  |  |  |
| No tumor | 94 (67.6) | 119 (75.3) | 0.126 |
| Resection organ | 44 (31.7) | 36 (22.8) |  |
|  |  |  |  |
| Ileum |  |  |  |
| No tumor | 87 (62.6) | 108 (68.4) | 0.488 |
| Removal tumor | 45 (32.4) | 41 (25.9) |  |
| Resection organ | 5 (3.6) | 6 (3.8) |  |
|  |  |  |  |
