# Supplementary material for: Adjuvant Use of PlasmaJet Device During Cytoreductive Surgery for Advanced-Stage Ovarian Cancer: Results of the PlaComOv-study, a Randomized Controlled Trial in The Netherlands
Source: Ann Surg Oncol. 2022 May 13;29(8):4833–43. doi: 10.1245/s10434-022-11763-2 (PMC9246793; doi:10.1245/s10434-022-11763-2)
Supplement: Supplementary file 3 — Supplementary file3 (DOCX 14 kb) [file 10434_2022_11763_MOESM3_ESM.docx]

Table S3: Subset-analysis, cytoreductive surgery with HIPEC procedure

|  | HIPEC | | | NON-HIPEC | | |
| --- | --- | --- | --- | --- | --- | --- |
|  | Intervention n=29 (%) | Control n=32 (%) | P.overall | Intervention n=110 (%) | Control n=129 (%) | P.overall |
| Surgical outcome |  |  | 0.106 |  |  | 0.011 |
| Complete | 28 (96.6) | 26 (81.2) |  | 91 (82.7) | 89 (69.0) |  |
| Optimal | 1 (3.5) | 6 (18.8) |  | 11 (10.0) | 32 (24.8) |  |
| Suboptimal | 0 | 0 |  | 8 (7.3) | 8 (6.2) |  |
|  |  |  |  |  |  |  |
| Operative time (mean, minutes) [SD] | 392 [135] | 372 [94] | 0.505 | 219 [87] | 193 [78] | 0.019 |
|  |  |  |  |  |  |  |
| Blood loss (mean, ml) [SD] | 1255 [866] | 1332 [737] | 0.713 | 950 [776] | 903 [797] | 0.646 |
|  |  |  |  |  |  |  |
| Hospitalization (days) [SD] | 10.5 [4.3] | 11.0 [8.2] | 0.780 | 8.7 [7.1] | 7.3 [5.9] | 0.085 |
| Intensive care (days) [SD] | 1.5 [1.1] | 1.7 [0.8] | 0.736 | 2.2 [2.4] | 1.5 [1.0] | 0.294 |
|  |  |  |  |  |  |  |
| Colostomy | 2 (6.9) | 8 (25.0) | 0.196 | 7 (6.4) | 12 (9.5) | 0.508 |
