# Supplementary material for: Adjuvant Use of PlasmaJet Device During Cytoreductive Surgery for Advanced-Stage Ovarian Cancer: Results of the PlaComOv-study, a Randomized Controlled Trial in The Netherlands
Source: Ann Surg Oncol. 2022 May 13;29(8):4833–43. doi: 10.1245/s10434-022-11763-2 (PMC9246793; doi:10.1245/s10434-022-11763-2)
Supplement: Supplementary file 4 — Supplementary file4 (DOCX 13 kb) [file 10434_2022_11763_MOESM4_ESM.docx]

Table S4: Sub-analysis, surgical outcome in case of peritoneal carcinomatosis (≥50 lesions)

|  | ≥50 lesions | |  | <50 lesions | |  |
| --- | --- | --- | --- | --- | --- | --- |
|  | **Intervention**  **n=54 (%)** | **Control n=66 (%)** | P.overall | **Intervention n=85 (%)** | **Control n=95 (%)** | P.overall |
| Complete cytoreduction |  |  | 0.034 |  |  | 0.135 |
| YES | 39 (72.2) | 34 (51.5) |  | 80 (94.1) | 82 (86.3) |  |
| NO | 15 (27.8) | 32 (48.5) |  | 5 (5.9) | 13 (13.7) |  |
