# Supplementary material for: Adjuvant Use of PlasmaJet Device During Cytoreductive Surgery for Advanced-Stage Ovarian Cancer: Results of the PlaComOv-study, a Randomized Controlled Trial in The Netherlands
Source: Ann Surg Oncol. 2022 May 13;29(8):4833–43. doi: 10.1245/s10434-022-11763-2 (PMC9246793; doi:10.1245/s10434-022-11763-2)
Supplement: Supplementary file 5 — Supplementary file5 (DOCX 13 kb) [file 10434_2022_11763_MOESM5_ESM.docx]

Table S5. Surgeons opinion regarding the added value of using the PlasmaJet during surgery per procedure

|  | n=139 (%) | |
| --- | --- | --- |
| PlasmaJet has not been used | | 32 (23) |
| PlasmaJet used, complete cytoreductive surgery was not possible | | 13 (9) |
| PlasmaJet used, but without PlasmaJet complete cytoreductive surgery would have been possible | | 35 (25) |
| PlasmaJet used, very useful to achieve complete cytoreductive surgery | | 40 (29) |
| PlasmaJet used, necessary to achieve complete cytoreductive surgery | | 16 (12) |
| Missing | | 3 (2) |
