# Supplementary material for: Adjuvant Use of PlasmaJet Device During Cytoreductive Surgery for Advanced-Stage Ovarian Cancer: Results of the PlaComOv-study, a Randomized Controlled Trial in The Netherlands
Source: Ann Surg Oncol. 2022 May 13;29(8):4833–43. doi: 10.1245/s10434-022-11763-2 (PMC9246793; doi:10.1245/s10434-022-11763-2)
Supplement: Supplementary file 6 — Supplementary file6 (DOCX 13 kb) [file 10434_2022_11763_MOESM6_ESM.docx]

|  | Experience PlasmaJet  1-10 procedures | |  | Experience PlasmaJet  11 or more procedures | |  |
| --- | --- | --- | --- | --- | --- | --- |
|  | **Intervention**  **n=52 (%)** | **Control n=59 (%)** | P.overall | **Intervention n=87 (%)** | **Control n=102 (%)** | P.overall |
| Complete cytoreduction |  |  | 0.256 |  |  | 0.013 |
| YES | 45 (86.5) | 45 (76.3) |  | 74 (85.1) | 70 (68.6) |  |
| NO | 7 (13.5) | 14 (23.7) |  | 13 (14.9) | 32 (31.4) |  |

Table S6. Surgical outcome related to experience in using the PlasmaJet
